# Also looking like *Limulus*? – retinula axons and visual neuropils of Amblypygi (whip spiders)

**DOI:** 10.1186/s12983-018-0293-6

**Published:** 2018-12-19

**Authors:** Tobias Lehmann, Roland R. Melzer

**Affiliations:** 1 Bavarian State Collection of Zoology – SNSB, Münchhausenstraße 21, 81247 Munich, Germany; 2 Ludwig-Maximilians-Universität München, Department Biologie II, Großhaderner Straße 2, 82152, Planegg-Martinsried, Germany; 3 GeoBioCenter LMU, Richard -Wagner-Str. 10, 80333 Munich, Germany

**Keywords:** Chelicerata, Arachnida, Visual system, Central projections, Phylogeny

## Abstract

**Background:**

Only a few studies have examined the visual systems of Amblypygi (whip spiders) until now. To get new insights suitable for phylogenetic analysis we studied the axonal trajectories and neuropil architecture of the visual systems of several whip spider species (*Heterophrynus elaphus*, *Damon medius*, *Phrynus pseudoparvulus*, and *P. marginemaculatus*) with different neuroanatomical techniques. The R-cell axon terminals were identified with Cobalt fills. To describe the morphology of the visual neuropils and of the protocerebrum generally we used Wigglesworth stains and μCT.

**Results:**

The visual system of whip spiders comprises one pair of median and three pairs of lateral eyes. The R-cells of both eye types terminate each in a first and a second visual neuropil. Furthermore, a few R-cell fibres from the median eyes leave the second median eye visual neuropil and terminate in the second lateral eye neuropil. This means R-cell terminals from the lateral eyes and the median eyes overlap. Additionally, the arcuate body and the mushroom bodies are described.

**Conclusions:**

A detailed comparison of our findings with previously studied chelicerate visual systems (i.e., Xiphosura, Scorpiones, Pseudoscorpiones, Opiliones, and Araneae) seem to support the idea of close evolutionary relationships between Xiphosura, Scorpiones, and Amblypygi.

## Background

The around 190 species of the arachnid order of Amblypygi (whip spiders or tailless whip scorpions) inhabit subtropical or tropical regions worldwide [1]. Most species occur in rain forests, only a few in savannahs or deserts, and all species are nocturnal predators. They can be found in leaf litter, underneath bark or stones, or in caves [2]. Fossil whip spiders are rare; the oldest specimens are known from the Late Carboniferous (ca. 310 Ma) [3, 4].

Their main sense organs are the antenniform legs (first pair of legs) and in particular their distal tarsi. These legs are no longer used for walking but are modified extremities carrying various sensilla (contact chemoreceptors, olfactory chemoreceptors, and mechanoreceptors) – very much like the antennae of insects [5–11]. Furthermore, the trichobothria and slit sense organs on the walking legs (2nd – 4th pair of legs) are important sensory structures [2, 12].

**Fig. 1:**
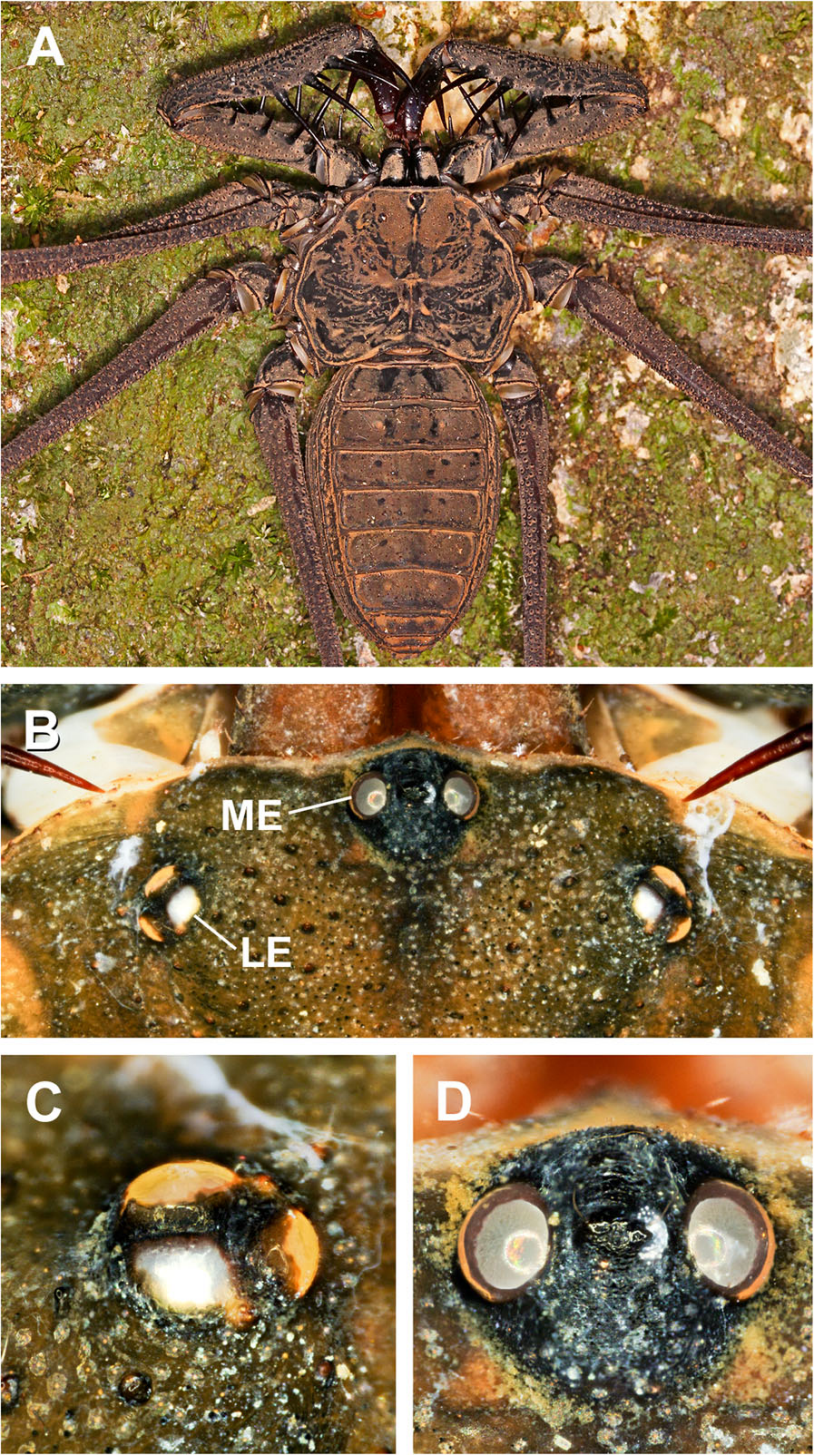
Median and lateral eyes in Amblypygi. *Heterophrynus elaphus* (**a**), *Damon medius* (**b**-**d**). **a**, Whip spider in natural habitat. **b**, Anterior part of the cephalothorax with one pair of median eyes and three pairs of lateral eyes. **c**, Detail of lateral eyes. **d**, Detail of median eyes

As nocturnal animals most activity of whip spiders takes place in darkness, hence the visual system certainly plays a subordinate role in spatial orientation [5]. Nevertheless, whip spiders have two classes of eyes: one pair of larger elevated eyes on the frontal region of the central carapace commonly referred to as median eyes, and one pair of three smaller eyes further back on each side of the lateral carapace, commonly referred to as lateral eyes (see Fig. 1). Little is known about the detailed structure of these eyes [2, 13–15]. The median eyes of whip spiders are formed from an invagination and are everted, hence the rhabdomeres point towards the light, and the nucleus lies basally [2, 15]. A tapetum, which reflects the incoming light towards the rhabdomeres, is absent. For instance in *Phrynus marginemaculatus,* there are slightly more than 100 R-cells (or retinula cells) per median eye [2]. The lateral eyes of whip spiders develop from a thickening of the epithelium without invagination. These eyes are of the inverted type, hence the rhabdomeres are located proximally and the nuclei distally to the incoming light [2, 15]. The lateral eyes have a reflecting tapetum [2]. For instance, in *P. marginemaculatus,* there are about 75 R-cells per lateral eye [2].

The eyes probably measure light intensity and play a role in adjusting the circadian rhythm [2]. Whip spiders, for example, react to sudden illumination and find dark places in which to hide quickly [2]. Even less is known about the neuroanatomy of the visual systems. So far, the only studies on the visual neuropils are of Hanström [16], Weygoldt [2], and Wiegmann*,* et al. [6]. Hanström reported two successive median eye neuropils and one lateral eye neuropil in *Neophrynus.* Weygoldt tentatively noted two visual neuropils for both median and lateral eyes. Finally, Wiegmann et al. superficially demonstrate the presence of the higher order neuropils, mushroom bodies and arcuate body, but could not find distinct visual input.

Despite the problems in resolving the phylogenetic tree of Chelicerata (see the recent review by Giribet [17]), the relationships of Amblypygi seem to be clear. Together with Thelyphonida, Schizomida and Araneae they form the clade Tetrapulmonata (the orders typically with four lungs), well supported by molecular and morphological data [18–22]. However, the placement of Tetrapulmonata within the chelicerate tree is difficult. Modern molecular and novel morphological data support a relationship of Tetrapulmonata with Scorpiones forming the clade Arachnopulmonata – endorsed by the homology of the book lungs of scorpions with the lungs of tetrapulmonates [23–25]. One alternative theory is, e.g., a sister group relationship of Tetrapulmonata with Palpigradi [19, 21, 26].

In order to fill the substantial knowledge gap on whip spider visual systems, and to find new morphological data to support or refute one of the current phylogenetic hypotheses, in the present paper we analyse the visual system of four amblypygid species (*Heterophrynus elaphus*, *Damon medius, Phrynus marginemaculatus,* and *P. marginemaculatus*). We used various neuroanatomical techniques (Cobalt fills, Wigglesworth stains, μCT, and AMIRA-3D-reconstruction) to locate the target neuropils of the axon terminals of the R-cells and describe the number, form, connectivity and general morphology of the visual neuropils. Furthermore, the higher order neuropils arcuate body and mushroom bodies are described. These neuropils probably also play a role in the integration of visual information in chelicerates [27–29]. By now, the visual systems of several chelicerate orders have been studied [28–39]. Therefore, comparable morphological data on the visual systems of Pycnogonida, Xiphosura, Scorpiones, Pseudoscorpiones, and Opiliones is available and can be compared to our recent findings in Amblypygi.

## Results

### General layout of the visual system and of the protocerebrum

The visual system in the studied amblypygid species is composed of one pair of median eyes located medially on the front corner of the cephalothorax, and one pair of three lateral eyes located laterally on each side of the cephalothorax (Fig. 1). The nerve fibres project from the median and lateral eyes proximally to the lateral protocerebrum.

**Fig. 2:**
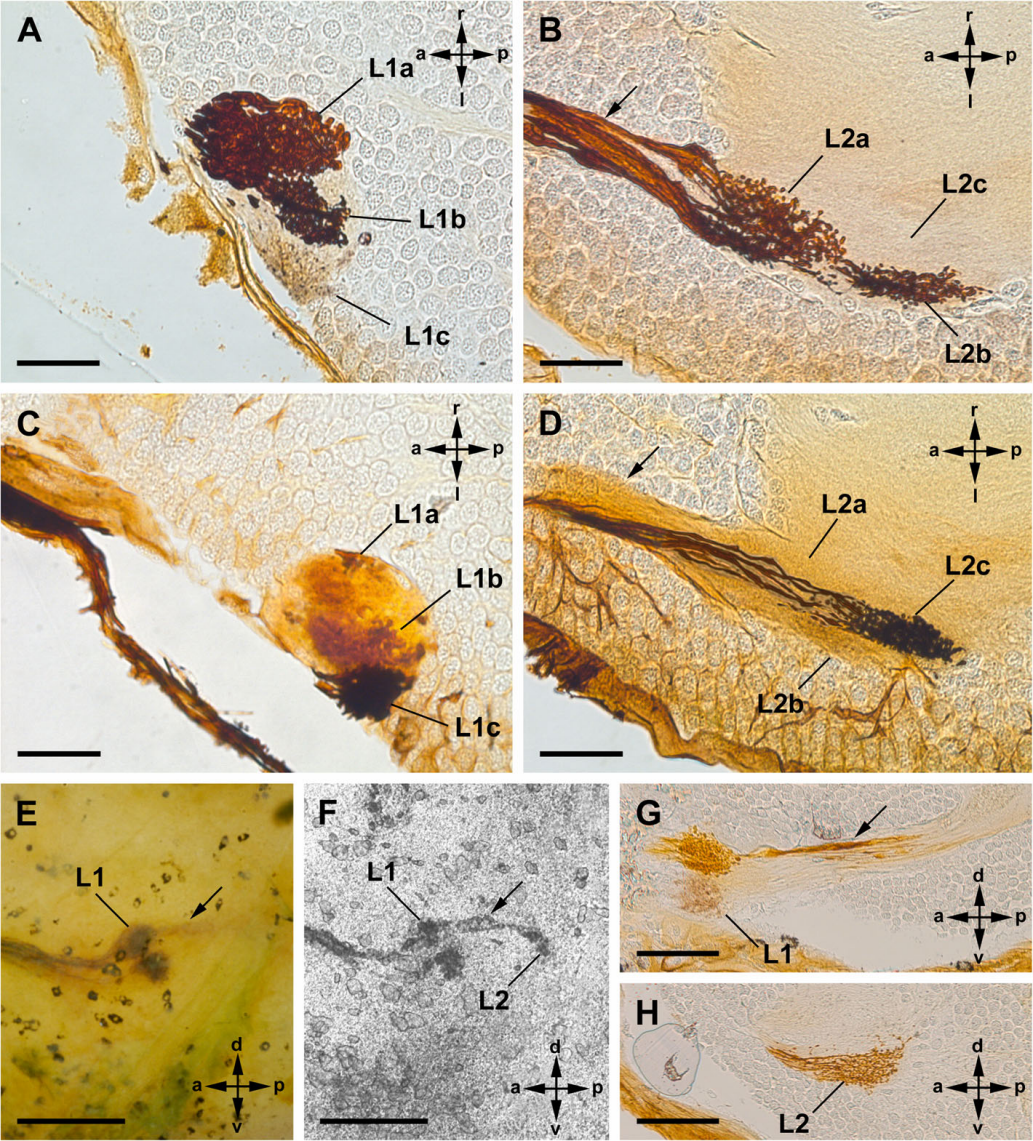
Cobalt fills via lateral eyes in Amblypygi. *Damon medius*; frontal sections (**a**-**d**); sagittal view (**e**-**h**). **a**, **b**, Cobalt fills via two lateral eyes, accordingly two subunits (subunit a, b) of first (**a**) and second (**b**) visual neuropils are stained. Note dense arrangement with varicosities of Cobalt-filled profiles. In the tract between the first and second neuropil (arrow) it seems, that the fibres cross. Bars 50 μm. **c**, **d**, Cobalt fills via one lateral eye (different eye than in **a**, **b**), accordingly different subunit (subunit c) of the first (**c**) and second (**d**) visual neuropil are stained than in **a**, **b**. Bars 50 μm. **e-h**, Comparison of whole mount, μCT, and sections of same specimen. Two lateral eyes are filled with cobalt, hence two subunits of the first lateral eye visual neuropil are stained. Dorsally the first and second visual neuropil is stained with cobalt, but ventrally cobalt stopped in the first visual neuropil, and the second neuropil remained unstained. In whole mount (**e**; bar 250 μm) only the first visual neuropil is definitely visible. In μCT reconstruction (**f**; bar 250 μm) the first and second neuropil is visible. Most details become apparent in sections (**g**, **h**; bars 100 μm). L1, first lateral eye neuropil; L2, second lateral eye neuropil; **a-c** subunits of L1 and L2; arrow, tract connecting L1 and L2

The three lateral eyes are connected to two distinct, successive visual neuropils as targets of the R-cell axons (Fig. 2). The first lateral eye neuropil is located in a ventrolateral part of the protocerebrum, underneath the first visual neuropil of the median eyes (see below). It is oval and embedded in the cell body rind of the brain, and subdivided into three subunits, one for each lateral eye. The second neuropil lies posterior to the first neuropil in a central position of the protocerebrum. It is also subdivided into three subunits, one for each lateral eye (Fig. 2).

**Fig. 3:**
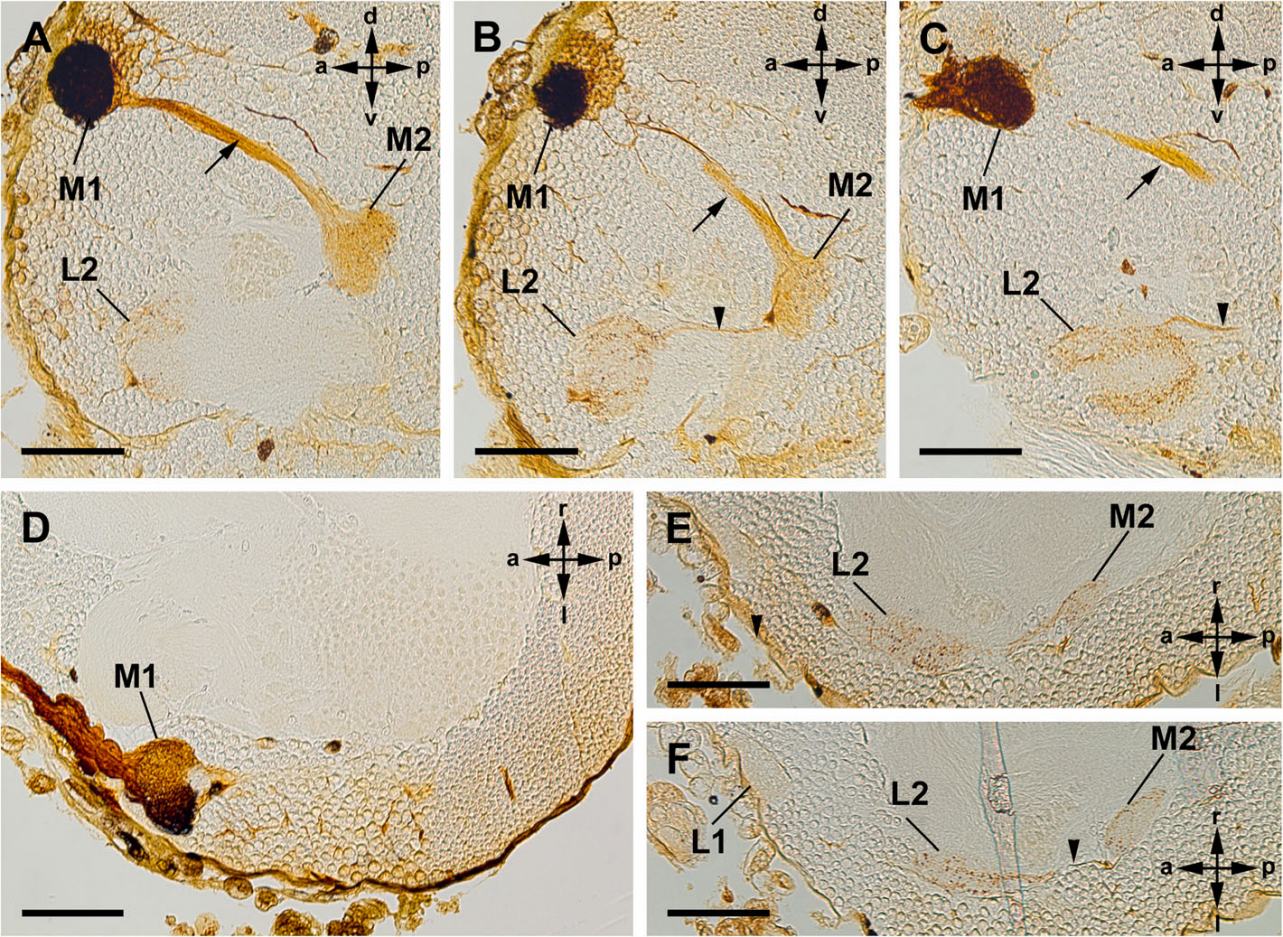
Cobalt fills via median eyes in Amblypygi. *Phrynus marginemaculatus*; sagittal sections (**a**-**c**); frontal sections (**d**-**f**). **a**, **b**, Two consecutive sections of the same specimen. **a**, First and second median eye visual neuropil filled with cobalt. Note dense arrangement of Cobalt-filled profiles in the first visual neuropil and just a few filled fibres running further to the second visual neuropil resulting in a diffuse staining of the neuropil. Both neuropils are connected via a tract (arrow). Bar 100 μm. **b**, First and second visual neuropil stained. Additionally, a thin tract connects the second median eye neuropil and the second lateral eye neuropil (arrowhead). A few cobalt filled R-cell axons run through this tract and terminate in the second lateral eye neuropil. Bar 100 μm. **c**, Same situation in a different specimen. Some cobalt filled R-cells from the median eye terminate in the second lateral eye visual neuropil. Bars 100 μm. **d**-**f**, Same situation in a third specimen and in frontal sections. Bars 100 μm. M1, first median eye visual neuropil; M2, second median eye visual neuropil; L1, first lateral eye neuropil; L2, second lateral eye neuropil; arrow, tract connecting M1 and M2; arrowhead thin tract connecting M2 and L2

**Fig. 4:**
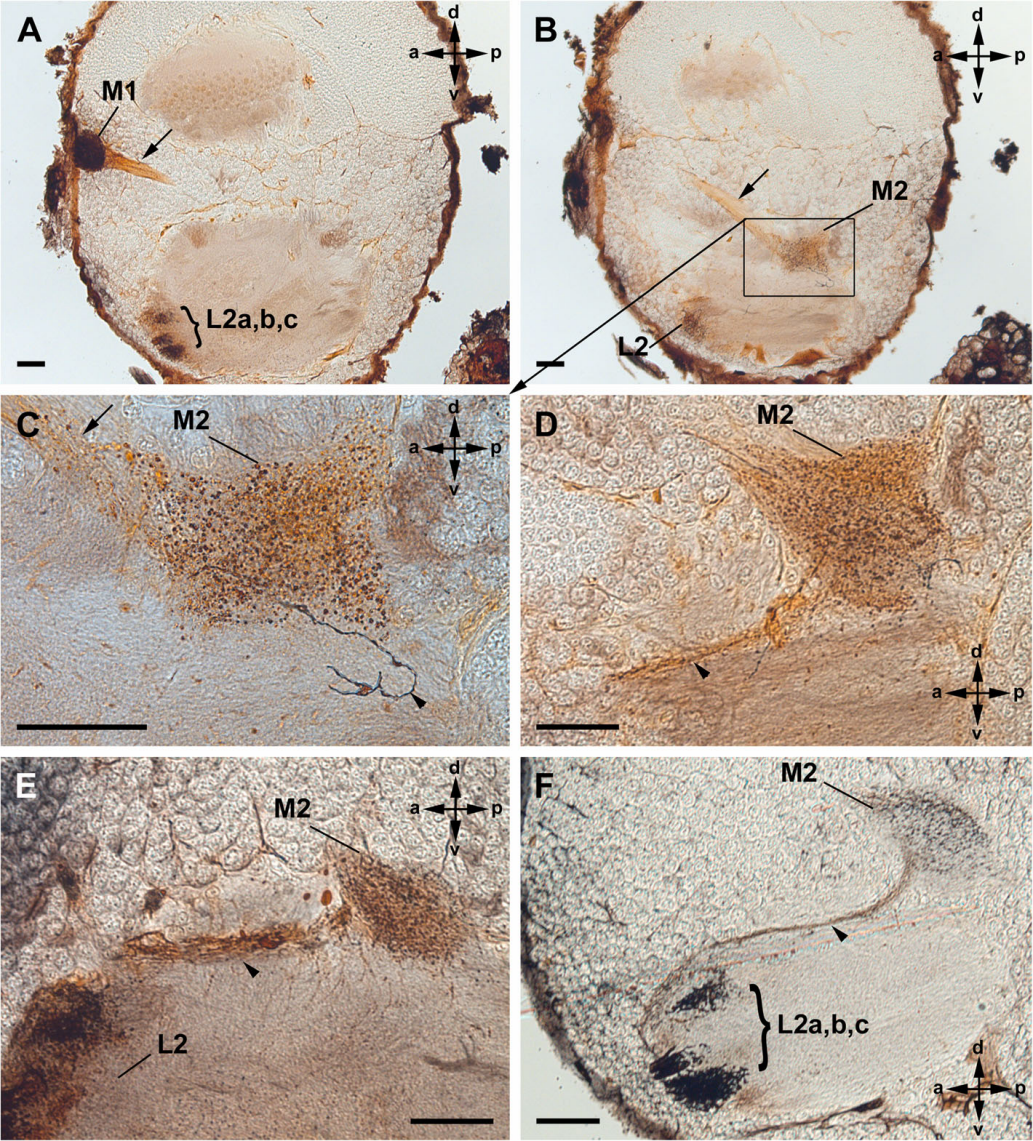
Cobalt fills simultaneously via median and lateral eyes in Amblypygi. *Phrynus pseudoparvulus*; all eight eyes stained; sagittal sections. **a**-**d**, Three consecutive sections of the same specimen; **c** detail of **b**. Cobalt visible in (I) the first median eye visual neuropil, (II) the tract between first and second median eye neuropil (arrows), (III) the second median eye visual neuropil, (IV) a thin tract between the second median eye and second lateral eye visual neuropil (arrowheads), (V) the second lateral eye neuropil, (VI) the tract between the first and second median eye neuropil (not shown, but available on morph-d-base) and (VII) the first lateral eye neuropil (not shown, but available on morph-d-base). Note U-turn of a few fibres (arrowheads) from the second median eye neuropil to a thin tract connecting the second median and second lateral eye neuropil. Bars 50 μm. **e**, **f**, Different specimens showing details of the connection between the second median and second lateral eye neuropil via a thin tract (arrowhead). Bars 50 μm. M1, first median eye visual neuropil; M2, second median eye visual neuropil; L2, second lateral eye neuropil; arrow, tract connecting M1 and M2; **a-c** subunits of L2; arrowhead thin tract connecting M2 and L2

The two median eyes also supply two distinct, successive visual neuropils as targets of the R-cell axons (Figs. 3, 4). The first neuropil is located in a dorsolateral part of the protocerebrum. The neuropil is spherical and embedded in the cell body rind of the brain. The second neuropil lies deeper, under the cell body rind and in a more ventral and posterior position in the protocerebrum. Furthermore, a few fibres leave the second median eye neuropil and end in the second lateral eye neuropil. Hence, axonal trajectories of the median and lateral eyes end in a shared region.

**Fig. 5:**
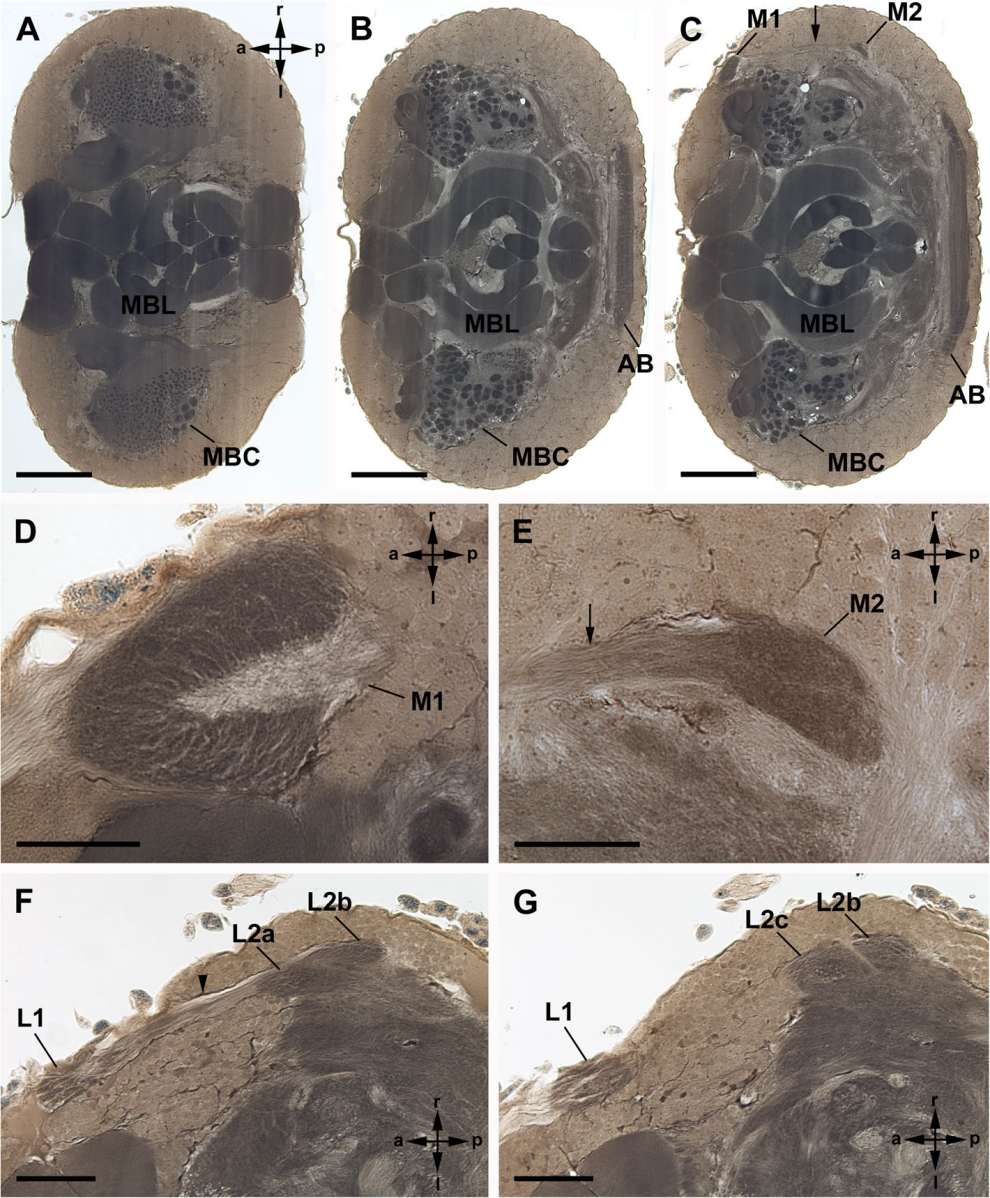
General anatomy of visual neuropils and protocerebrum in Amblypygi, frontal sections. *Heterophrynus elaphus*; Wigglesworth stains. **a**-**c**, Three sections from dorsal (**a**), central (**b**) and more ventral (**c**) part of the protocerebrum showing large-scale spread of the mushroom bodies, especially of the mushroom body lobes. Note the relatively small arcuate body. Bars 250 μm. **d**, **e**, Detail of the first and second median eye visual neuropil. Bars 50 μm. **f**, **g**, Detail of the first and second lateral eye visual neuropil. Note subdivision of the second visual neuropil into three subunits. Bars 100 μm. AB, arcuate body; MBC, mushroom body calyces; MBL, mushroom body lobes; M1, first median eye visual neuropil; M2, second median eye visual neuropil; L1, first lateral eye neuropil; L2, second lateral eye neuropil; arrow, tract connecting M1 and M2; **a-c** subunits of L2; arrowhead tract connecting L1 and L2

**Fig. 6:**
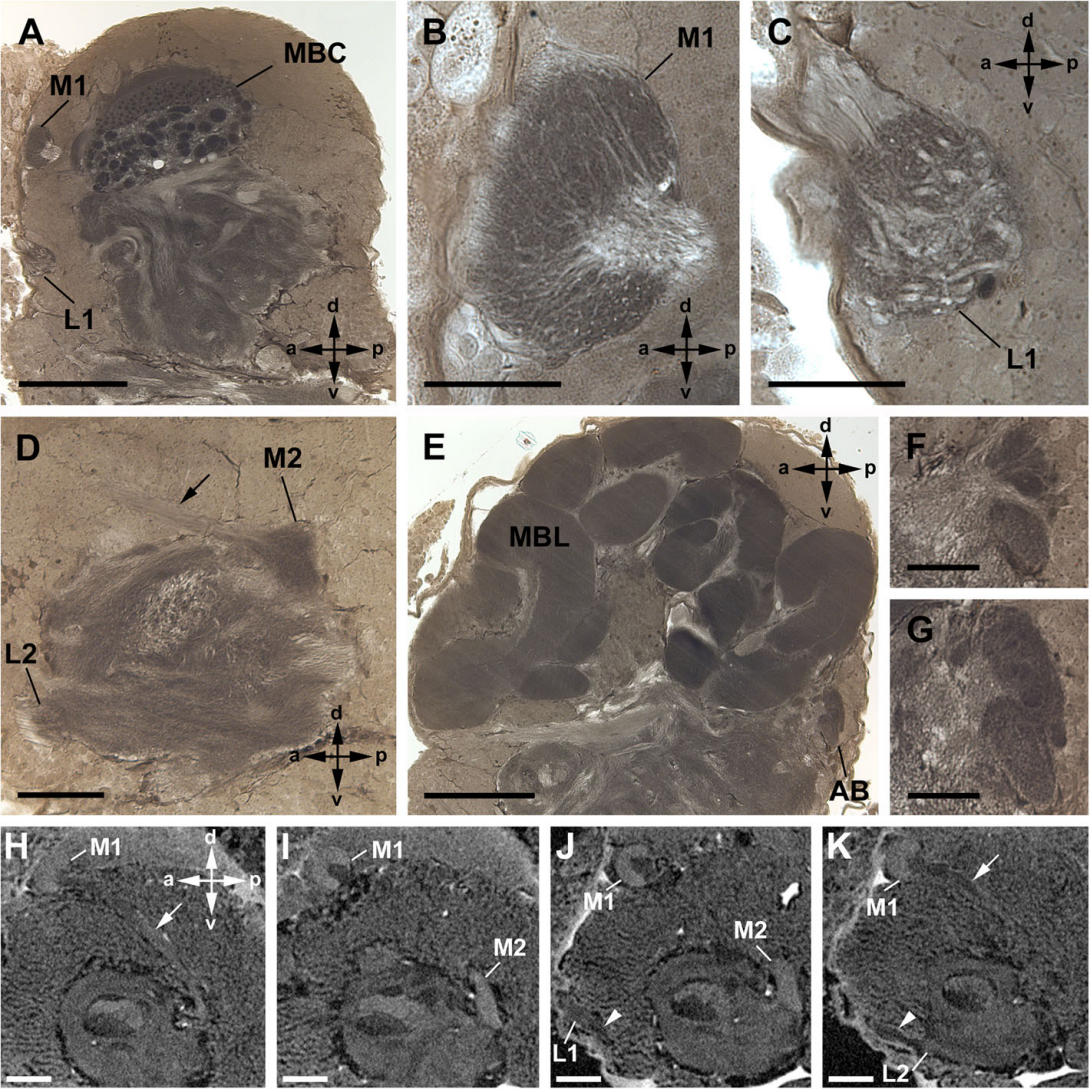
General anatomy of visual neuropils and protocerebrum in Amblypygi, sagittal sections. *Heterophrynus elaphus* (**a-g**) and *Damon medius* (**h**-**k**); Wigglesworth stains (**a-g**) and μCT (**h**-**k**, additional raw data on morph-D-base). **a**, Dorsal to the first median eye visual neuropil lies the first lateral eye neuropil. Bar 250 μm. **b**, **c**, Detail of the first median and lateral eye visual neuropil. Bars 50 μm. **d**, Section showing a tract connecting the first and second median eye neuropil, the second median eye neuropil, and the second lateral eye neuropil. Bar 100 μm. E, Mid-sagittal section showing the mushroom body lobes filling almost the entire protocerebrum. Note the relatively small arcuate body. Bar 250 μm. **f**, **g**, Detail of the arcuate body in parasagittal (**f**) and midsagittal (**g**) region. Bars 50 μm. **h**, **i**, μCT reconstruction showing the first and second median eye visual neuropil and a tract (arrow) connecting these two neuropils. Bars 250 μm. **j**, **k**, μCT reconstruction showing the first and second lateral eye visual neuropil and a tract (arrow) connecting these two neuropils. Bars 250 μm. AB, arcuate body; MBC, mushroom body calyces; MBL, mushroom body lobes; M1, first median eye visual neuropil; M2, second median eye visual neuropil; L1, first lateral eye neuropil; L2, second lateral eye neuropil; arrow, tract connecting M1 and M2; arrowhead tract connecting L1 and L2

The visual neuropils are unequivocally identified with Cobalt fills, and also can be recognised with μCT scans and in Wigglesworth stains, as dark-stained areas, as is typical for dense neuropils such as sensory neuropils (Figs. 5, 6).

Furthermore, the arcuate body is found in a posterior position in the ventral protocerebrum; its shape is slightly bent anteriorly. The mushroom bodies are located parallel to the midline on each side of the dorsal protocerebrum. Both neuropils can be recognised with Wigglesworth stains and μCT (Figs. 5, 6).

### Lateral eye visual neuropils

Cobalt fills via the lateral eyes reveal two distinct retinula axon target regions in each hemisphere of the protocerebrum, a first and a second visual neuropil (Fig. 2).

After entering the first visual neuropil, the retinula axons build synaptic varicosities all over their extensions (Fig. 2a, c). The first visual neuropil is subdivided into three subunits. When three eyes are cobalt filled, all three subunits are stained. Accordingly, when two eyes are filled, two subunits are stained and one filled eye results in one stained subunit (Fig. 2a, c). A chiasma between the first and second visual neuropils is not positively identified in any of the chosen section planes (sagittal, frontal, or transversal) and in any method (cobalt fills, Wigglesworth stains, or μCT). However, when seen from a frontal view, fibres of the three eyes are observed that seem to cross each other between the first and second visual neuropil, but it rather seems that the fibres from each eye are twisted and do not build a “classical” chiasma as in Tetraconata (Fig. 2b). In the second visual neuropil, the retinula axon terminals are branched and have synaptic varicosities (Figs. 2b, d, 4a, b, e, f). Here, also three subunits receiving the trajectories of one of the three eyes each are visible. As in the first neuropil, when three eyes are cobalt-filled, all three subunits are stained. Accordingly, when two eyes are filled two subunits are stained and one filled eye results in one stained subunit (Figs. 2b, d; 4a, f). Both visual neuropils are also visible in μCT and Wigglesworth stains (Figs. 5f, g; 6a, c, d, j, k). When comparing the results in *D. medius* and *P. pseudoparvulus* – the two species where cobalt fills via the lateral eyes were made – we found no species-specific differences. We also found no differences, when comparing the neuroanatomy of these two species with the Wigglesworth stains in *H. elaphus*.

### Median eye visual neuropils

Cobalt fills via the median eyes revealed three distinct retinula axon target regions in each hemisphere of the protocerebrum: the first and second median eye visual neuropil, and additionally a few R-cell axons even reach to the second lateral eye neuropil (Figs. 3 and 4).

Immediately after entering the brain, the retinula axons build synaptic varicosities in a crescent-shaped region of the first visual neuropil (Figs. 3a-d, f; 4a). This region is also visible in Wigglesworth stains as darker stained region and in μCT scans (Figs. 5c, d; 6a, b, h-k). Proximal to the crescent-shaped region the visual fibres are concentrated and the retinula axons project ventro-posteriorly in a tract through the cell body rind deeper into the protocerebrum (Figs. 3a-c; 4a-c; 5c, e; 6d, h). Again, a chiasma between the first and second visual neuropils is not positively identified. Most of these fibres end in the second median eye visual neuropil. In the cobalt fills, only a diffuse staining and a few slightly darkened fibres are visible, indicating that only a few R-cell axons terminate in the second visual neuropil (Figs. 3a, b, e, f; 4b-f). The second median eye visual neuropil is also visible in μCT scans and Wigglesworth stains (Figs. 5c, e; 6d, i, j). Even fewer fibres leave the second neuropil and point in a thin tract anteriorly and terminate in the second lateral eye neuropil (Figs. 3b, c, e, f; 4c-f). Again, in the cobalt fills, only a diffuse staining and a few slightly darkened fibres are visible, indicating that only a few R-cell axons are stained. The cobalt filled R-cells from the median eyes are found throughout the whole second lateral eye neuropil (Figs. 3b, c, e, f). The thin tract that connects the second median and second lateral eye visual neuropil is only visible in cobalt fills and not in μCT scans and Wigglesworth stains. As in the lateral eyes, we found no species-specific differences between the studied species (*P. pseudoparvulus*, *P. marginemaculatus*, and *H. elaphus*).

### Arcuate body

The arcuate body is rather small (Figs. 5b, c; 6e-g). It is found in a more ventral position of the posterior protocerebrum. The arcuate body is composed of two layers and slightly bent anteriorly. A direct visual input of R-cells is not observed. However, tracts from the second lateral and second median eye neuropils seem to point towards the arcuate body.

### Mushroom bodies

The mushroom bodies are exceptionally large (Figs. 5a-c; 6a, e). Altogether, the mushroom body allocates about two-thirds of the neuropil of the protocerebrum. The mushroom body lobes are folded several times (Figs. 5a-c, 6e). In the midsagittal region, the lobes occupy almost the whole protocerebrum (Figs. 5a-c, 6e). The mushroom body calyces are also large. They are subdivided into glomeruli of two different kinds (Figs. 5a-c, 6a). Dorsally one-third of the calyces consist of small glomeruli (diameter 5–9 μm) and laterally two-thirds of the calyces of large glomeruli (diameter 20–30 μm). A direct (R-cells) or indirect (tracts from the visual neuropils) visual input is not observed.

## Discussion

### Comparison with previous literature

So far, the only studies concerning the visual neuropils of Amblypygi were published by Hanström [16], Weygoldt [2], and Wiegmann*,* et al. [6]. Comparing the present results with the remarkable study of Hanström, one finds many accordances, but also some discrepancies. He studied the visual neuropils of the whip spider *Neophrynus sp.*. For the median eyes, he reported a first large neuropil (“Sehmasse”) in the peripheral part of the brain and a less distinct second neuropil at the edge of the neuropil mass. This reflects our results very well. However, the few median eye R-cell fibres that leave the second median eye neuropil and terminate in the second lateral eye neuropil are not mentioned by Hanström. For the lateral eyes, he described one shared neuropil only. We showed clearly that the lateral eyes also have two successive visual neuropils. Furthermore, he characterised as we did a small arcuate body and giant mushroom bodies*.* Weygoldt [2] just mentioned briefly two visual neuropils (“optic masses”) for each median eye and for each lateral eye group. However, his statements are without further illustrations, what makes it difficult for comparison. Finally, Wiegmann*,* et al. [6] looked for visual input into higher order neuropils and stated that there is no visual input into the mushroom bodies or arcuate body.

In the present study, we unequivocally identified for the first time the R-cell terminals of the median and lateral eyes of whip spiders by using cobalt as a precise marker, a tool Hanström did not have at that time. From this, the visual neuropils are characterized. Furthermore, we describe the general neuroanatomy of the protocerebrum based on classical histology and μCT.

Our main findings are: (I) The R-cells of the lateral eyes are connected to a first and a second lateral eye visual neuropil. Both neuropils are subdivided into three subunits, one for each lateral eye. (II) The R-cells of the median eyes are connected to a first and a second median eye visual neuropil. Furthermore, (III) a few median eye R-cell fibres leave the second median eye neuropil and terminate in the second lateral eye neuropil. This means that some R-cells from the median eyes and from the lateral eyes terminate in a shared region, i.e. the second lateral eye visual neuropil. (IV) The two-layered arcuate body is relatively small compared to (V) the giant mushroom bodies. These are composed of several times folded mushroom body lobes and mushroom body calyces with two different kinds of glomeruli (small and large ones).

When interpreting the results we must consider what kind of neuropil the second lateral eye neuropil might be. With a few R-cells from the median eyes terminating herein, is it possible that it is a median eye neuropil as well? Alternatively, could it even be a higher order neuropil or “pre-integration centre” where information from both eye types is interconnected? Our interpretation is that it is a lateral eye neuropil, because the vast majority of the input comes from the lateral eyes, and just a few fibres come from the median eye. One possible function of this interconnection from both eye types could be lateral inhibition [40].

Furthermore, it deserves mention here that we found no species-specific differences in the four studied species. This corresponds to the general assumption that the nervous system displays a high degree of conservatism. This, in turn, makes comparative studies on the structure and development of the nervous systems suitable for higher-level phylogenetic analysis, e.g. the phylogeny of the chelicerate orders. For this field of research two different approaches – “neurophylogeny” [41–43] and “neural cladistics” [44, 45] – were established.

### Comparison with other chelicerates

The features described in the present study allow a comparison with the visual systems in other chelicerates. In all studied chelicerate visual systems, for both median eyes and lateral eyes, one constant feature appeared – two successive visual neuropils with direct input from the eyes via R-cell terminals [30, 32, 33, 35, 38, 39]. However, the only exception is Araneae (spiders). Here, for both eye types, just the first visual neuropils receive direct input and in the second neuropils, no R-cell terminals are found [28, 29].

Hence, in the forefront of this study, the question arose, if whip spiders, follow the rule of two neuropils and do they have short and long R-cell fibres as, e.g., horseshoe crabs, scorpions, harvestmen, and pseudoscorpions, or just short fibres, as described for the spider *Cupiennius salei*?

**Fig. 7:**
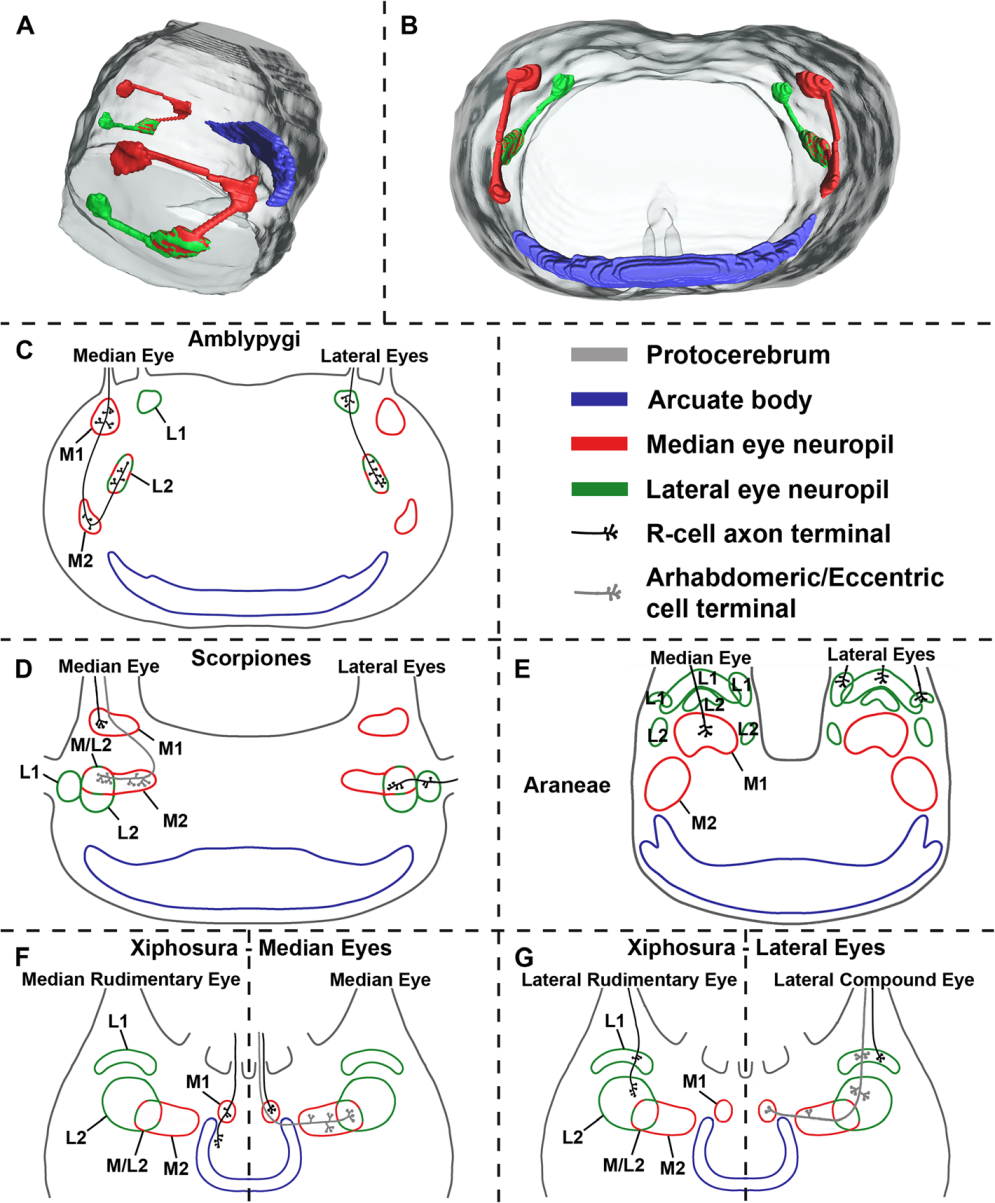
Comparison of median and lateral eye visual systems in (**c**) Amblypygi, (**d**) Scorpiones, (**e**) Araneae, and (**f**, **g**) Xiphosura. **a**, **b**, 3D serial reconstruction of the visual system of whip spider *Heterophrynus elaphus*. **c**, Amblypygi (*Damon medius, Heterophrynus elaphus, Phrynus spp.*), this study. **d**, Scorpiones (*Euscorpius spp., Androctonus australis*), after Lehmann*,* et al. [33] and Fleissner [40]. **e**, Araneae *(Cupiennius salei)*, after Strausfeld*,* et al. [28], Strausfeld*,* et al. [29]. **f**, **g**, Xiphosura *(Limulus polyphemus)*, after Lehmann*,* et al. [33], Calman*,* et al. [38], Chamberlain*,* et al. [39]. L1, first lateral eye visual neuropil; L2, second lateral eye visual neuropil; M1, first median eye visual neuropil; M2, second median eye visual neuropil; M/L2, region were M1 and L1 overlap

As shown above, it became apparent, that amblypygids do not merely have for both – median eyes and lateral eyes – two successive visual neuropils with direct R-cell input, but also show striking similarities with horseshoe crabs and scorpions (Fig. 7). In all three chelicerate orders – Xiphosura, Scorpiones, and Amblypygi – R-cell fibres from the median eyes terminate in the second lateral eye neuropil. This means that in all three, there is a region where median and lateral eye R-cell terminals overlap.

In the lateral rudimentary eyes (not the lateral compound eyes) of the horseshoe crab (*Limulus polyphemus*), the R-cells terminate in the first and second lateral eye neuropil (Fig. 7g). In the median eyes (not the median rudimentary eyes), the photoreceptor cells terminate in the first median eye visual neuropil and the arhabdomeric cells (non-photosensitive, secondary neurons in the retina) terminate in a second median eye neuropil that partly overlaps with the second lateral eye neuropil (Fig. 7f) [36–39, 46]. Hence, there is a region, where R-cell terminals of median and lateral eyes overlap (see discussion in Lehmann and Melzer [33]). In scorpions, the second visual neuropils overlap as well, or rather the R-cell fibres from the median (also the arhabdomeric cells) and from the lateral eyes (Fig. 7d). Lehmann*,* et al. [33] already discussed the close evolutionary relationships of the visual systems in Scorpiones and Xiphosura (median eyes and lateral rudimentary eyes).

Although they are less numerous, than, e.g., in scorpions, we also found in whip spiders R-cell fibres from the median eyes, that terminate in the second lateral eye neuropil (Fig. 7c). One difference is that in the studied amblypygid species, these fibres are found throughout the whole second lateral eye neuropil and not – as in *Limulus* and scorpions – in a part of the neuropil. Furthermore, for *Limulus* and scorpions, it seems that the second median and lateral eye neuropils overlap. This is not the case in whip spiders. Here the second visual neuropils are connected via a thin tract, where the median eye R-cells run through.

One question that emerges concerns the nature of these fibres from the median eyes that terminate in the second lateral eye neuropil. In *Limulus* and scorpions, these long, overlapping fibres are from the arhabdomeric cells. So far, only one detailed TEM-study on the eyes of Thelyphonida (whip scorpions), the sister taxon of Amblypygi exists [47]. The structure of the eyes of Amblypygi and Thelyphonida seem to be similar [2, 47]. Meyer-Rochow [47] described arhabdomeric cells for both median and lateral eyes in the whip scorpion *Thelyphonus caudatus* but had difficulties to determine their exact location and number. Hence, if whip spiders also possess arhabdomeric cells, it is possible, that these fibres could have their origin in the arhabdomeric cells. However, further investigation on the arhabdomeric cells in whip spiders and on the visual neuropils of whip scorpions is needed.

The other arachnid orders differ. In Araneae – the only other studied arachnid order with both median and lateral eyes (some mites (Acariformes) do also have both eye types) – different numbers of visual neuropils are reported in different spider species [16, 28, 29, 48, 49]. For the median eyes (principal eyes or anterior median eyes), a first and a second visual neuropil are always described. However, for the lateral eyes (secondary eyes) the situation differs. Generally speaking, for most web-spinning spiders (e.g., Araneidae, Deinopidae, Pholcidae) only one lateral eye neuropil and for free living or active hunting spiders (e.g., Ctenidae, Salticidae, Lycosidae) two (first and second) lateral eye neuropils are reported [16, 49, 50]. In *Cupiennius* – the best studied spider species – the R-cells from the median and lateral eyes terminate in the first median and the first lateral eye visual neuropil, respectively, and no long R-cell fibres to the second visual neuropils are described (Fig. 7e) [40, 41]. In Opiliones and Pseudoscorpiones, having either median or lateral eyes, this overlapping naturally cannot be found [16, 32, 35, 51, 52].

## Conclusions

Before we started our series of studies about the visual systems in various chelicerates [30–35], of which the present one is the most recent, the number of visual neuropils seemed to be inconsistent. Thus, e.g., in scorpions one neuropil was described for the median eyes and three for the lateral eyes [16], in harvestmen, which have just median eyes, three neuropils [51], in pseudoscorpions, which have just lateral eyes, one neuropil [16, 53], and in whip spiders two for the median eyes and either one or two for the lateral eyes [2, 16]. Furthermore, in Pycnogonida two visual neuropils were reported, but the suggested second order neuropil was displaced into a different position [54]. Conversely, it now becomes apparent that in all chelicerate visual systems, we studied until now, regardless of whether median eyes or lateral eyes, one constant feature appeared – two successive visual neuropils [28–30, 32, 33, 35, 38–40]. This type of architecture is well known from other arthropods, e.g. from Myriapoda (having just lateral eyes) [55, 56] and from the lateral eyes in Tetraconata (crustaceans and insects) (reviewed, e.g., in [57–59]), and might be plesiomorphic for the euarthropod main lineages. According to Tanaka*,* et al. [60] even in fossil Megacheira (great appendage arthropods) – which strongly resemble early chelicerates – two successive visual neuropils are found. Furthermore, at the current state of our survey, in all chelicerate orders, the first and the second order neuropils receive direct input from the eyes via R-cell terminals – with one exception, Araneae.

The phylogenetic interpretation of these findings is difficult. Are the similarities of the visual systems between Xiphosura, Scorpiones, and Amblypygi just functionally caused, conditioned by the fact that they all have median and lateral eyes, or are they also a phylogenetic signal for the suggested clade Arachnopulmonata – consisting of Araneae, Amblypygi, Thelyphonida and Scorpiones [25]? To formulate it cautiously our findings are certainly no counterevidence against close relationships between these four lineages. However, more research on the central projections of the R-cells and the visual neuropils in Thelyphonida and Araneae is necessary.

Why does Araneae not fit into the concept? Spiders also have median and lateral eyes and their sister group relationships to Amblypygi and Thelyphonida forming together the clade Tetrapulmonata seems to be clear [18–22], but their neuroanatomy seems to be a bit confused. For the lateral eyes, only one or two neuropils are reported and for both eyes types, just fibres terminating in the first visual neuropils are proven [16, 28, 29, 49]. One explanation would lie in the methodology. Probably, the second lateral eye neuropil was overlooked in the histological sections of these studies and would be only visible in TEM, as in Pseudoscorpiones or Opiliones [32, 35]. Concerning the R-cell projections, Hanström [16] used osmium stains and Strausfeld*,* et al. [28, 29] Golgi impregnation and Reduced silver staining. Hence, for them it was probably not possible to study the exact projection of the R-cells. On that account, unequivocal tracers like the cobalt fills used in this study should be applied in spiders. Another explanation for the differences in the neuroanatomy could be that in the evolution of the visual system of this mega-diverse order some reductions took place. Probably, in the web spinning spiders the visual system became less important, what might have resulted in the reduction of the second lateral eye neuropil. Finally, in spiders arhabdomeric cells are not known. Arhabdomeric cells typically bear distal dendrites that contact R-cells and, therefore, they are considered to play a role as secondary neurons in the processing of visual information. So far, only photoreceptor cells and non-pigmented supportive cells or glia, but no arhabdomeric cells are described from the retina of spiders (see, e.g., [61–66]). Hence, a connection from the arhabdomeric cells to the second visual neuropil – as in horseshoe crabs, scorpions, and whip spiders – would be missing.

## Material & Methods

### Animals

Specimens of *Heterophrynus elaphus* Pocock, 1903 were collected during a field trip to the Private Protected Area ACP Panguana in Peru (collecting permit #007–2014-SERFOR-DGGSPFFS, export permit # 0001757-SERFOR). Specimens of *Damon medius* (Herbst, 1797) and *Phrynus marginemaculatus* Koch, 1841 were obtained from bugzuk.com. Specimens of *Phrynus pseudoparvulus* Armas & Viquez, 2001 were obtained from vogelspinnen-kauf.de.

### Cobalt fills

Modified after Altman*,* et al. [67]: Specimens (overall 38 specimens, therefrom 22 successfully stained: *Damon medius,* median or lateral eyes filled, *n* = 13, *Phrynus pseudoparvulus,* median and lateral eyes filled simultaneously, *n* = 6; and *Phrynus marginemaculatus,* median eyes filled, *n* = 3) were anaesthetized by using CO_2_. CoCl_2_ crystals were inserted into the median, lateral, or median and lateral eyes with a fine tungsten needle. After diffusion times between 5 and 22 h, cobalt was precipitated with a solution of five drops of (NH_4_)_2_S in 10 ml H_2_O_dest_. After fixation of the cephalothorax in AAF (85 ml 100% ethanol, 10 ml 37% formaldehyde, 5 ml glacial acetic acid), the brain was dissected. The brain was silver intensified as follows: 60 min at 50 °C in the dark in solution A (10 ml H_2_O_dest_, 3 ml 100% ethanol, 0.5 g gum arabic, and 0.02 g hydroquinone; pH value adjusted to between 2.6 and 2.8 using citric acid), and 20–40 min at 50 °C in the dark in solution B (10 ml H_2_O_dest_, 3 ml 100% ethanol, 0.5 g gum arabic, 0.02 g hydroquinone, 0.01 g AgNO_3_; pH value adjusted to between 2.6 and 3.1 using citric acid). Silver intensification was stopped in an acetic acid solution (50 ml 30% ethanol, 5 g glucose, pH value adjusted to between 2.6 and 3.1 using acetic acid). After dehydration in a graded acetone series, the specimens were embedded in Glycidether 100, and sectioned with a rotary microtome and stainless steel blade in the sagittal and frontal planes (14–16 μm). Finally, some sections were silver intensified in solution A and B for a second time.

### Wigglesworth stains (osmium-ethyl-gallate procedure)

Modified after Wigglesworth [68], Leise*,* et al. [69], Mizunami*,* et al. [70]: Specimens (*Heterophrynus elaphus*, *n* = 6) were anaesthetized by using CO_2_ followed by decapitation. Brains were dissected and fixed overnight in 4% glutardialdehyde in 0.1 M cacodylate buffer at 4 °C. After postfixation in 1% OsO_4_ in 0.1 M cacodylate buffer (2 h at 4 °C) animals were stained for 20 h at 4 °C in a saturated ethyl gallate solution, dehydrated in a graded acetone series, embedded in Glycidether 100, and sectioned with a rotary microtome and stainless steel blade in the sagittal and frontal planes (10–12 μm).

### μCT

We tried, for the first time, to find out whether or not Cobalt fills can be seen in the μCT. The μCT reconstruction is compared to whole mounts and sections and thus provides another line of evidence complementing the results from histological sections. The results are shown in Fig. 2.

Modified after Sombke*,* et al. [71]: Micro-CT scanning of the brain without cobalt fills (*Damon medius*, *n* = 2): Specimens were anaesthetized by using CO_2_ followed by decapitation. Brains were dissected and fixed overnight in 4% formaldehyde in phosphate buffer (Roti®-Histofix 4%, Carl Roth GmbH + Co. KG, Germany) and dehydrated in a graded ethanol series. After incubation in 1% iodine solution in 100% ethanol for 48 h and several washing steps in 100% ethanol, the brains were critical-point-dried.

Micro-CT scanning of the brain with cobalt fills (*Damon medius*, *n* = 1): Cobalt filled brain (see above) was stored and scanned in 30% ethanol.

Scanning was performed with a Phoenix Nanotom M (GE Measurement & Control, Wunstorf, Germany) cone beam CT scanner at a voltage of 90 kV and a current of 170 mA (critical point dried brain without cobalt fills, voxel size 1,58 μm) or 100 kV and 120 mA (cobalt filled brain in 30% ethanol, voxel size 1,82 μm), using a standard target. 1440 projections were prepared per scan. The 3D datasets (prepared with the datos|x reconstruction software, GE Measurement & Control) were examined by VGStudioMax 2.2 (Visual Graphics GmbH, Heidelberg, Germany) software.

### 3D-reconstruction

The specimen (*Heterophrynus elaphus*, prepared as for Wigglesworth stains) was cut into a complete transversal series (12 μm). Slices were mounted on glass slides, covered with cover-slips, and photographed under a conventional light microscope. Images were contrast-enhanced in Adobe Photoshop, then aligned, segmented and rendered in Amira.
